# Impact of successful treatment with direct-acting antiviral agents on health-related quality of life in chronic hepatitis C patients

**DOI:** 10.1371/journal.pone.0205277

**Published:** 2018-10-09

**Authors:** Regina Juanbeltz, Iván Martínez-Baz, Ramón San Miguel, Silvia Goñi-Esarte, Juan Manuel Cabasés, Jesús Castilla

**Affiliations:** 1 Department of Pharmacy, Complejo Hospitalario de Navarra, Pamplona, Spain; 2 Instituto de Salud Pública de Navarra—IdiSNA, Pamplona, Spain; 3 CIBER Epidemiología y Salud Pública (CIBERESP), Pamplona, Spain; 4 Department of Gastroenterology, Complejo Hospitalario de Navarra, Pamplona, Spain; 5 Department of Economics, Public University of Navarra, Pamplona, Spain; Centers for Disease Control and Prevention, UNITED STATES

## Abstract

**Background:**

Direct-acting antivirals (DAA) have demonstrated high efficacy to achieve sustained virological response (SVR) in chronic hepatitis C patients. We aim to assess the change in health-related quality of life (HRQoL) among patients successfully treated, and to identify predictors of this variation.

**Methods:**

In a prospective observational study, patients with chronic hepatitis C who started DAA therapy between May 2016 and April 2017 completed the EQ-5D-5L questionnaire at baseline and 12 weeks after the end of therapy before knowing the virological result. Analysis included all patients with SVR.

**Results:**

Median baseline EQ-5D-5L scores of the 206 enrolled patients were 0.857 utility and 70.0 visual analogue scale (VAS). Following SVR, a reduction occurred in the proportion of patients with mobility problems (35% vs 24%, p = 0.012), pain/discomfort (60% vs 42%, p<0.001) and anxiety/depression (57% vs 44%, p = 0.012), with an increase in utility (+0.053, p<0.001) and VAS (+10, p<0.001). Score improvements were also observed in cirrhotic (+0.048 utility, p = 0.027; +15 VAS, p<0.001) and HIV co-infected patients (+0.039 utility, p = 0.036; +5 VAS, p = 0.002). In multivariate analyses, middle age (45–64 years) and baseline anxiety/depression were associated to greater improvement in utility after SVR, and moderate-advanced liver fibrosis and cirrhosis to greater increase in VAS score. Low baseline values were associated to greater improvements in utility value and VAS score.

**Conclusions:**

The cure of chronic hepatitis C infection with DAA has a short term positive impact on HRQoL with improvement in mobility, pain/discomfort, anxiety/depression, utility value and VAS score. Patients with poor baseline HRQoL were the most beneficed.

## Introduction

Chronic hepatitis C constitutes a well-recognize global public health issue, mainly due to its worldwide high prevalence and the serious consequences of progression of disease. Hepatitis C virus (HCV) leads to cirrhosis in up to 20% of those chronically infected [1] and of these, 2–4% annually develop hepatocellular carcinoma [2]. Moreover, chronic HCV infection is the primary indication for liver transplantation in developed countries [3]. The economic burden is multiplied by the impact of HCV on health related quality of life (HRQoL), appreciable at any stage of severity [4,5]. Complications of advanced liver disease such as encephalopathy, variceal hemorrhage, and ascites have been reported to negatively affect HRQoL [6,7]. Extrahepatic manifestations related to HCV as fatigue, irritability, depression, muscle pain, joint pain and cognitive impairment may also influence the patient's psychological well-being and self-perceived health [8,9].

Measuring HRQoL has become important in clinical research, as it is considered the gold standard to report the patient´s experiences with illness and treatment [10,11]. Changes in HRQoL from the patient perspective before and after health care interventions can be monitorized with instruments like the EQ-5D [12], a standardized questionnaire that provides a simple, generic measure of health for clinical and economic appraisal [13]. EQ-5D provides health utilities, widely used in cost-effectiveness and decision analyses where different treatments are compared.

Antiviral therapies can eradicate the HCV resulting in improvements in liver histology, morbidity, mortality and enhancing HRQoL because of symptoms´ alleviation [14]. A combination of pegylated interferon (peg-IFN) and ribavirin (RBV) has long been the standard treatment for chronic hepatitis C, with limited efficacy and significant adverse effects [15]. The impact of peg-IFN on quality of life was studied in patients with and without HIV co-infection, showing negative patient experiences [16–17]. However, patients who achieved HCV clearance experienced significant improvements in HRQoL compared to non-responders [17–19].

New direct acting antiviral regimens (DAA) have changed the landscape of treatment of chronic hepatitis C. DAA provide important advantages, including higher efficacy, shorter duration of treatment and an optimal safety profile [20–22]. However, there is little data regarding the effect of the new regimens on HRQoL and most of the evidence comes from clinical trials, in which certain subpopulations are underrepresented, such as psychiatric patients, those coinfected with HIV or those with addictive behaviours [23,24]. Assessment of HRQoL in real life from chronic hepatitis C patients is needed to evaluate the economic and health impact of these new therapies [25].

The main objective of this study was to assess short-term changes in HRQoL and health utilities among chronic hepatitis C patients receiving successful treatment with DAA. Secondary objectives were to analyse the influence of treatment in domains of EQ-5D-5L questionnaire, and to identify the predictors of change in HRQoL after HCV clearance.

## Patients and methods

### Design and study population

A prospective observational study was conducted in a regional reference hospital in northern Spain. Patients with chronic HCV infection who started interferon-free treatments with DAA between May 2016 and April 2017 were invited to participate. Exclusion criterion was inability to understand Spanish.

In accordance with the clinical practice guidelines and the centre's treatment protocol, patients started therapy with a variable duration of 8 to 24 weeks. Three interviews were conducted by the same investigator. The first was face-to-face, providing information to the patient and obtaining the written informed consent. Successive interviews were carried out by telephone in week 4 of treatment and 12 weeks after its completion (post-12), time when the sustained virologic response (SVR) is assessed, revealing the cure or not of the HCV infection. Patients were considered to have achieved SVR if they had undetectable HCV-RNA at post-12 week. The last interview was conducted before the follow-up medical visit, so that the patient and the interviewer were still unaware of the analytical data and treatment's outcome.

According to the main objective of the study, only those patients with the three questionnaires completed and who had achieved SVR were included in the analysis.

The study fulfilled all the ethical requirements and was approved by the Clinical Research Ethics Committee of Navarre.

### Assessments

Sociodemographic variables of the patient were recorded at the baseline interview: sex, age, marital status, educational level and occupational status. Clinical variables were obtained from the electronic medical record: alcohol consumption and parenteral drug use history, smoking, body mass index and concomitant diseases, which were assessed using a simplified Charlson index [26] and the diagnosis count method [27]. In addition, the existence of HIV co-infection, hypertension, rheumatological disease, paralysis/hemiplegia, depression, anxiety or other psychiatric illness was specifically collected.

Finally, the variables related to chronic liver disease due to HCV were included: viral genotype, previous treatment experience (naïve, pre-treated with IFN or peg-IFN, pre-treated with DAA), degree of liver fibrosis based on Fibroscan test's values, categorized according to cut-off points F0-F1 (<7.5 kPa), F2 (7.5 to 9.4 kPa), F3 (9.5 to 12.4 kPa) and F4 (≥12.5 kPa), Child-Pugh score in the cirrhotic patient [28], current combination of DAA, and duration of treatment.

### HRQoL questionnaire

All patients were asked to complete the Spanish version of the EQ-5D, an instrument previously used to assess HRQoL in patients with chronic hepatitis C [6,29]. Currently, there are two versions of the EQ-5D of the EuroQol Group, EQ-5D-3L and EQ-5D-5L. In this study the new version was used, the EQ-5D-5L, that has proven to be valid and more sensitive to changes in health status [30].

EQ-5D-5L provides a simple description of the patient's self-perceived health status covering five health dimensions: mobility, self-care, usual activities, pain/discomfort and anxiety/depression, with five response options (no problems, slight problems, moderate problems, severe problems and extreme problems). Any response in the items “slight, moderate, severe or extreme” was considered as having “problems”, for each dimension. The questionnaire also provides a self-reported Visual Analogue Scale (VAS), which measures the patients' health on a scale from 0 to 100, where 0 reflects the worst imaginable health status and 100 the best imaginable health status. The patient response in the five dimensions results in a five-digit code, which can be transformed to a single measure called EQ-5D index or utility value. It ranges from 0 (reference value assigned to death) to 1 (perfect health), with the possibility of negative values for health states considered worse than death [31]. It is a health summary score used in the clinical and economic evaluation of healthcare as well as in population health surveys [32]. Although the EuroQol Group has already developed a methodology for eliciting value sets for the 5L version in some countries, no EQ-5D-5L value set was available in Spain at the moment of the study. Therefore, following the EuroQol recommendation, an interim mapping method or crosswalk to obtain 5L value sets from the existing 3L values for Spain was used in this study (available at https://euroqol.org/eq-5d-instruments/eq-5d-5l-about/valuation-standard-value-sets/crosswalk-index-value-calculator/).

### Statistical analysis

Frequencies and proportions were calculated for the categorical variables, and the mean and standard deviation for continuous variables. For each interview, the proportion of patients with problems in each of the EQ-5D-5L questionnaire dimension was calculated, and the median of the utility and the VAS score was estimated. The differences in the medians of these indices were compared using the Wilcoxon signed rank test. The comparison was repeated in strata by age group, degree of liver fibrosis, HIV co-infection, simplified Charlson index, duration of treatment, RBV use, considering baseline utility and baseline VAS score above or below the median. Multivariable linear regression analyses were carried out to identify factors associated with change in HRQoL, with their β coefficients and 95% confidence intervals (CIs). The differences between post-12 and baseline scores in utility value and in VAS score were respectively the outcome variables. A positive β coefficient indicated improvement and a negative coefficient decline in utility or VAS score after SVR. The adjusted models included sex, age groups (<45; 45–64 and ≥65 years), HIV co-infection, baseline limitation of mobility, baseline anxiety-depression disorders and degree of liver fibrosis (F0-F1, F2-F3 and F4) before hepatitis C treatment.

## Results

### Characteristics of patients

A total of 271 patients started treatment during the study period, 214 of those signing the informed consent (79%). Eight patients were excluded from the final analysis, 4 due to loss during the follow-up and another 4 due to not achieving SVR, leaving 206 patients cured of chronic HCV infection. The average age was 52.3 years (SD = 9.0) and 66% were males. HIV co-infected patients represented 32% of the population. The simplified Charlson score was ≥2 in 22 patients (11%) and 90% had at least one comorbidity, the most frequent being a psychiatric disorder (34%), hypertension (23%) and rheumatological diseases (12%). A total of 66% patients were smokers, 16% had a history of alcohol abuse and 52% had a history of parenteral drug use as probable mode of HCV transmission (Table 1).

**Table 1:** Socidemographic and clinical characteristics of the patients.

Characteristics	Total (n = 206)
Age in years, mean (SD)	52.3 (9.0)
Male sex, n (%)	135 (66)
Marital status, n (%)	
Single	70 (34)
Married/Partnered	90 (44)
Divorced	36 (18)
Widow/Widower	10 (5)
Educational level, n (%)	
No education	11 (5)
Primary	83 (40)
Secondary	91 (44)
University	20 (10)
Not reported	1 (0.5)
Occupational status, n (%)	
Employed	101 (49)
Unemployed	63 (31)
Disabled	29 (14)
Retired	13 (6)
History of parenteral drug use, n (%)	107 (52)
HIV co-infection, n (%)	65 (32)
Comorbidity: Charlson index, n (%)	
0–1	184 (89)
2	14 (7)
≥ 3	8 (4)
Total number illnesses, mean (SD)	2.1 (1.4)
Body mass index, mean (SD) ^a^	25.8 (4.9)
HCV Genotype, n (%)	
1a	81 (39)
1b	49 (24)
2	7 (3)
3	43 (21)
4	24 (12)
Other	2 (1)
Prior HCV treatment experience, n (%)	
Naïve	163 (79)
Interferon failure	41 (20)
Direct acting antiviral failure	2 (1)
Duration of HCV treatment, n (%)	
8 weeks	20 (10)
12 weeks	148 (72)
> 12 weeks	38 (18)

Abbreviations: SD, standard deviation; HIV, human immunodeficiency virus; HCV, hepatitis C virus; RNA, ribonucleic acid; IQR, interquartile range.

^a^Body mass index is in Kg/m^2^.

The most frequent HCV genotypes were 1a (39%), 1b (24%) and 3 (21%). Liver fibrosis degree was F0-F1 in 51 (25%) patients, F2-F3 in 97 (47%) and F4 in 58 (28%) patients. Among 95% of cirrhotic patients had compensated liver disease (Child-Pugh A).

DAA combinations used were ombitasvir/paritaprevir/ritonavir +/- dasabuvir (39%), sofosbuvir/ledipasvir (33%), sofosbuvir + daclatasvir (22%), grazoprevir/elbasvir (3%), sofosbuvir/velpatasvir (1.5%), sofosbuvir (1.5%) and sofosbuvir + grazoprevir/elbasvir (0.5%). The therapeutic regimen included RBV in 52% of the cases, and 72% of the treatments lasted 12 weeks. A majority of the patients (79%) had never received previous treatment for hepatitis C (Table 1).

### EQ-5D-5L health dimensions

**Fig 1:**
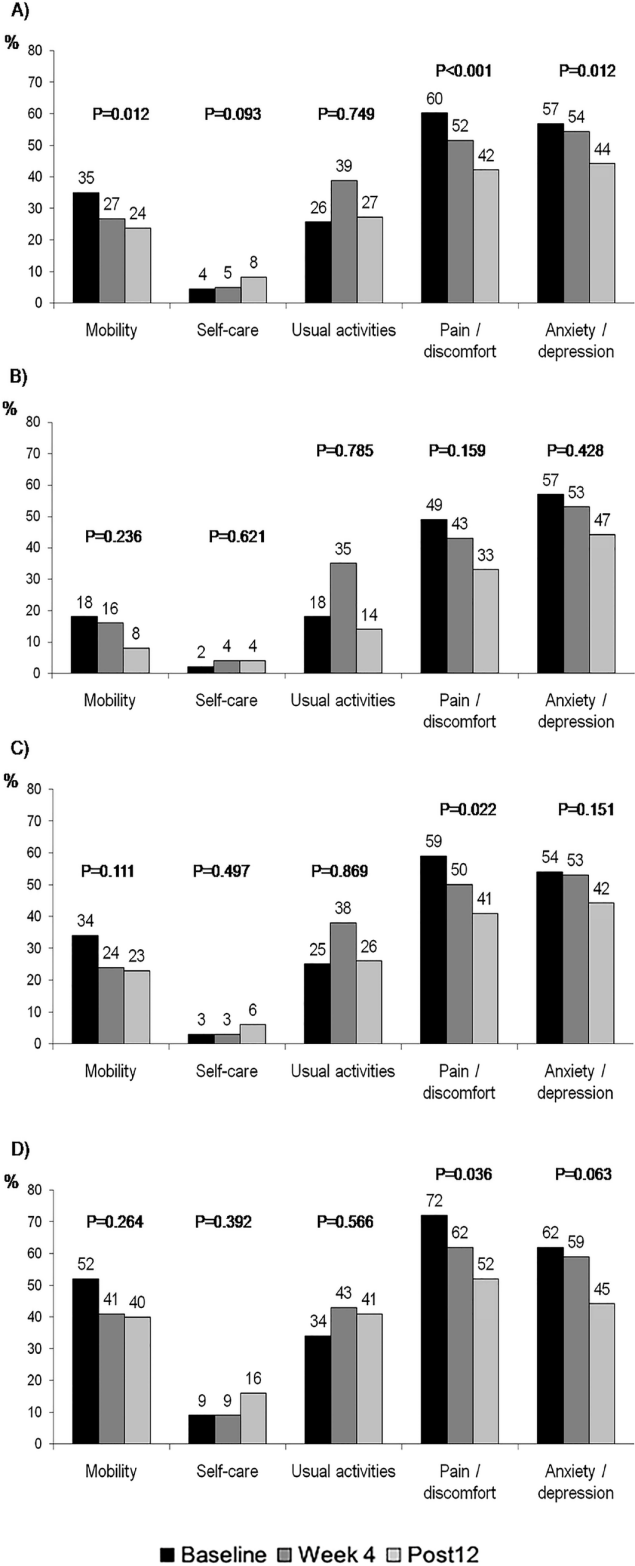
Percentage of patients reporting problem in any EQ-5D dimension by liver fibrosis stage and study visit. P values are the comparison of the proportions from baseline and week 12 post-treatment and were calculated by Chi-square test. (A) Total of study population; (B) F0-F1 patients; (C) F2-F3 patients; (D) Cirrhotic patients.

Prior to the start of treatment, the dimensions in which the patients showed a greater incidence of problems were pain/discomfort (60%), anxiety/depression (57%) and mobility (35%). The treatment started having a positive impact on these dimensions from week 4, and on all liver fibrosis subgroups (Fig 1). The baseline and post-12 comparison of the EQ-5D-5L questionnaire’s dimensions showed a decrease in the proportion of patients with mobility problems (35% vs 24%, p = 0.012), pain/discomfort (60% vs 42%, p<0.001) and anxiety/depression (57% vs 44%, p = 0.012). A statistically non-significant deterioration was observed in the dimensions of self-care and daily activities (Fig 1, panel A). Patients F0-F1 did not experience changes statistically significant after treatment (panel B), but improvement on pain/discomfort happened in F2-F3 (p = 0.002; panel C). Cirrhotic patients reported more problems for all dimensions before treatment, but improvements after therapy occurred in pain/discomfort (72% vs 52%, p = 0.036) and in the anxiety/depression dimension (62% vs 45%, p = 0.063), although the latter did not reach statistical significance (panel D).

### Utilities (EQ-5D-5L Index) and VAS score

The median utility was not affected during treatment (0.857, p = 0.324), while the VAS score showed a 5-point increase, from 70 baseline points to 75 in week 4 (p = 0.049). Poor baseline scores were associated to significant increases (Table 2).

**Table 2:** Changes in health-related quality of life scores from the baseline to the week 4 of treatment with direct-acting antiviral agents.

** **	Utility value				Visual analogical scale score			
	Baseline	Week 4	Difference	p value^a^	Baseline	Week 4	Difference	p value^a^
Total (n = 206)	0.857	0.857	0.000	0.324	70.0	75.0	5.0	0.049
Age, in years								
< 45 (n = 24)	0.884	0.856	-0.028	0.322	75.0	75.0	0.0	0.792
45–64 (n = 165)	0.857	0.857	0.000	0.404	70.0	70.0	0.0	0.038
≥ 65 (n = 17)	0.857	0.857	0.000	0.044	75.0	80.0	5.0	0.529
Liver fibrosis								
F0-F1 (n = 51)	0.893	0.871	-0.022	0.704	75.0	75.0	0.0	0.420
F2-F3 (n = 97)	0.857	0.871	0.014	0.324	70.0	75.0	5.0	0.053
F4 (n = 58)	0.809	0.822	0.013	0.325	60.0	67.5	7.5	0.041
HIV co-infection								
Yes (n = 65)	0.871	0.887	0.016	0.797	75.0	70.0	-5.0	0.706
No (n = 141)	0.857	0.857	0.000	0.329	70.0	75.0	5.0	0.039
Comorbidity								
Charlson 0–1 (n = 184)	0.857	0.857	0.000	0.399	70.0	75.0	5.0	0.084
Charlson ≥2 (n = 22)	0.784	0.838	0.054	0.702	50.0	60.0	10.0	0.304
Current Rivabirin use								
Yes (n = 106)	0.850	0.840	-0.010	0.276	70.0	70.0	0.0	0.373
No (n = 100)	0.857	0.887	0.030	0.019	70.0	77.5	7.5	0.057
Baseline utility								
≥ median (n = 108)	0.914	0.910	-0.004	0.001	80.0	80.0	0.0	0.540
< median (n = 98)	0.696	0.783	0.087	0.001	50.0	60.0	10.0	0.005
Baseline VAS score								
≥ median (n = 123)	0.910	0.910	0.000	0.141	80.0	80.0	0.0	0.018
< median (n = 83)	0.719	0.799	0.080	0.004	50.0	60.0	10.0	<0.001

Abbreviations: HIV, human immunodeficiency virus; HCV, hepatitis C virus; VAS, visual analogical scale.

^a^P value obtained by Wilcoxon’s test for repeated measures.

In comparison with the baseline value, the utility in post-12 week increased by 6% (+0.053, p<0.001), and the VAS increased 10 points (+14%, p<0.001). These improvements were statistically significant in cirrhotic patients (+0.048 utility, p = 0.027, +15 VAS points, p<0.001) and F2-F3 fibrosis subgroup (+0.053 utility, p = 0.002, +10 VAS points, p<0.001), but not in those F0-F1 (+0.039 utility, p = 0.061; +5 VAS points, p = 0.148). Analyses stratified by HIV co-infection, Charlson index and baseline scores showed important increases in the post-12 week utility or VAS score (Table 3).

**Table 3:** Changes in health-related quality of life scores from the baseline to the week 12 post-treatment with direct-acting antiviral agents.

	Utility value				Visual analogical scale score			
	Baseline	Post-12	Difference	p value^a^	Baseline	Post-12	Difference	p value^a^
Total (n = 206)	0.857	0.910	0.053	<0.001	70.0	80.0	10.0	<0.001
Age, in years								
< 45 (n = 24)	0.884	0.892	0.008	0.962	75.0	80.0	5.0	0.100
45–64 (n = 165)	0.857	0.910	0.053	<0.001	70.0	80.0	10.0	<0.001
≥ 65 (n = 17)	0.857	0.857	0.000	0.286	75.0	80.0	5.0	0.139
Liver fibrosis								
F0-F1 (n = 51)	0.893	0.932	0.039	0.061	75.0	80.0	5.0	0.148
F2-F3 (n = 97)	0.857	0.910	0.053	0.002	70.0	80.0	10.0	<0.001
F4 (n = 58)	0.809	0.857	0.048	0.027	60.0	75.0	15.0	<0.001
HIV co-infection								
Yes (n = 65)	0.871	0.910	0.039	0.036	75.0	80.0	5.0	0.002
No (n = 141)	0.857	0.893	0.036	<0.001	70.0	80.0	10.0	<0.001
Comorbidity								
Charlson 0–1 (n = 184)	0.857	0.910	0.053	<0.001	70.0	80.0	10.0	<0.001
Charlson ≥2 (n = 22)	0.784	0.857	0.073	0.316	50.0	70.0	20.0	0.004
Current HCV treatment duration								
8 weeks (n = 20)	0.857	0.850	-0.007	0.532	75.0	80.0	5.0	0.312
12 weeks (n = 148)	0.857	0.910	0.053	<0.001	70.0	80.0	10.0	<0.001
> 12 weeks (n = 38)	0.840	0.878	0.038	0.052	60.0	80.0	20.0	<0.001
Current Rivabirin use								
Yes (n = 106)	0.850	0.910	0.060	<0.001	70.0	80.0	10.0	<0.001
No (n = 100)	0.857	0.902	0.045	0.014	70.0	80.0	10.0	0.001
Baseline utility								
≥ median (n = 108)	0.914	1.000	0.086	0.688	80.0	85.0	5.0	0.001
< median (n = 98)	0.696	0.820	0.124	<0.001	50.0	70.0	20.0	<0.001
Baseline VAS score								
≥ median (n = 123)	0.910	0.932	0.022	0.051	80.0	85.0	5.0	0.061
< median (n = 83)	0.719	0.843	0.124	<0.001	50.0	70.0	20.0	<0.001

Abbreviations: HIV, human immunodeficiency virus; HCV, hepatitis C virus; VAS, visual analogical scale.

^a^P value obtained by Wilcoxon’s test for repeated measures.

Multivariable linear regression showed that age between 45–64 years (β = 0.07; 95%CI, 0.01 to 0.13) and suffering anxious or depressive disorders before starting treatment (β = 0.08; 95%CI, 0.03 to 0.12) were predictive factors of utility improvement after SVR and moderate-advanced liver fibrosis (β = 5.93; 95%CI, -0.01 to 11.86) and cirrhosis (β = 7.15; 95%CI, 0.64 to 13.65) favourable for increases in VAS score (Table 4).

**Table 4:** Effect of socio-demographic and clinical factors on post-12 health related quality of life improvement for sustained virological responders.

Baseline characteristics	Utility value^a^		Visual analogical scale score^b^	
	β coefficient (CI 95%)	P value	β coefficient (CI 95%)	P value
Intercept	-0.06 (-0.16 to 0.04)	0.248	-1.26 (-12.45 to 9.92)	0.824
Female sex	0.01 (-0.04 to 0.05)	0.802	2.06 (-3.07 to 7.20)	0.429
Aged 45–64 years vs <45yrs	0.07 (0.01 to 0.13)	0.041	1.81 (-5.34 to 8.96)	0.619
Aged ≥65 years vs <45yrs	0.07 (-0.02 to 0.17)	0.139	-1.46 (-12.15 to 9.22)	0.788
HIV co-infection	-0.02 (-0.06 to 0.03)	0.482	-2.92 (-8.08 to 2.24)	0.266
Limitation of mobility^c^	-0.04 (-0.10 to 0.02)	0.193	-4.70 (-11.18 to 1.77)	0.153
Anxiety/Depression	0.08 (0.03 to 0.12)	0.001	4.37 (-0.75 to 9.48)	0.094
F2-F3 vs F0-F1 liver fibrosis	0.03 (-0.02 to 0.08)	0.297	5.93 (-0.01 to 11.86)	0.050
F4 vs F0-F1 liver fibrosis	0.02 (-0.04 to 0.08)	0.422	7.15 (0.64 to 13.65)	0.031

Linear regression adjusted by sex, age groups, HIV co-infection, limitation of mobility, anxious-depression disorders and liver fibrosis.

Abbreviations: HIV, human immunodeficiency virus.

^a^Dependent variable is the difference post-12 minus baseline utility value.

^b^Dependent variable is the difference post-12 minus baseline visual analogical scale score.

^c^Limitation of mobility was defined by rheumatic disease, morbid obesity or paralysis/hemiplegia.

Poor baseline values of the utility and VAS score, when baseline values were introduced in their respective models, were the more predictive factors of improvement in the post-12 week in patients with SVR (β = -0.297; 95%CI, -0.403 to -0.190 and β = -0.459; 95%CI, -0.572 to -0.347, respectively).

## Discussion

Successful treatment with DAA on health-related quality of life in patients with chronic hepatitis C was associated to significant improvement in the majority of HRQoL domains measured by the EQ-5D-5L instrument. Improvement of HRQoL started shortly after the initiation of therapy and enhanced after achieving SVR, including cirrhotic and HIV co-infected patients, classically considered “difficult to treat” populations. Main improvements occurred in mobility, pain/discomfort and anxiety/depression dimensions, as well as in the health utility and VAS score. Although evidence from “real life” setting is lacking [23], patient reporting outcomes with DAA in clinical trials also suggest benefit after treatment [33–36]. Relationship between SVR and HRQoL improvement has not been elucidated, but viral clearance has been suggested to result in cytokines and inflammatory biomarkers reduction in periphery and central nervous system, leading to a positively impact on patients’ experience [37, 38]. The improvement observed in the well-being of patients cured with DAA reinforces the idea that chronic HCV infection, far from being a purely hepatic disease, presents a clearly systemic component and impaires HRQoL [39,40]. According the Spanish National Health Survey 2011/12 [41], the general population aged 45–54 years referred a VAS score of 77.2 and utility value of 0.928, being higher figures than those referred by our patients before treatment, 70.0 and 0.875, respectively. Lower scores in our patients suggest a HRQoL impairment associated to chronic hepatitis C infection [7, 29].

In the first 4 weeks of treatment, the median VAS score increased 5 points and improved health dimensions that were negatively affected with interferon treatments [42]. This early improvement in HRQoL has been described for DAA [35], contrary to what happened with peg-IFN + RBV and triple therapy with boceprevir or telaprevir [10,17,43,44], which seems to relate HRQoL deterioration during treatment to interferon and not to second generation DAA. The use of RBV was not associated with significant disutility, a fact that suggests a lower impact of side effects such as anaemia and pruritus, when administered in combination with DAA compared to when administered with interferon. This aspect reinforces the idea of the good tolerability of DAA in real life conditions [45,46].

In clinical practice, patient reporting outcomes constitute useful tools in the evaluation and monitoring of health interventions, since they provide information on patient perception and needs [47]. Knowing the predictors of HRQoL improvement after the treatment of hepatitis C can help to strengthen patient adherence and motivation. Middle age, baseline anxiety-depression, advanced fibrosis and cirrhosis were found to be the most consistent predictors of HRQoL improvements after SVR. Precisely, those factors have been previously associated with impairment in HRQoL among chronic hepatitis C patients [4,29,48] and appear to be key determinants for HRQoL deterioration prior to therapy [14]. The high prevalence of depression and anxiety in HCV patients before treatment initiation [49] would reinforce the need for psychosocial screening, even more when considering that a greater HRQoL improvement may occurs in those patients after SVR. In our study, patients with worse baseline HRQoL score showed more significant improvements, a common finding in all types of clinical trials [50]. This result is consistent with the greater improvement in HRQoL in cirrhotic patients, with comorbidity or HIV co-infection, observed in the stratified analysis of the study.

This study provides useful information for cost-effectiveness analysis. Utility values allow to obtain quality adjusted life years, and to estimate incremental cost-utility ratios in pharmacoeconomic analysis. Since the advent of second-generation DAA, some cost-utility analyses have been published to assess their efficiency [51–54], with more or less favourable incremental cost-utility ratios depending on the degrees of liver fibrosis, the cost of the drugs and the previous treatment experience. More favourable treatment efficiency in patients with a high degree of fibrosis is derived from the potential more imminent progression to severe stages, associated with greater disutility and greater consumption of health resources [51,52]. According to our results, the more significant HRQoL improvement in cirrhotic patients after the SVR reinforces this idea. In any case, a decline in the cost of drugs and the possibility of currently treating patients with a low degree of liver fibrosis with short 8-week regimens, would reduce the incremental cost-utility ratios and improve the efficiency of treatment in all patient subpopulations.

This real-life prospective study included hepatitis C patients who received treatment in a regional reference hospital in northern Spain. Although the epidemiology of HCV infection and the introduction of DAA have been relatively homogeneous in Spain, we can not rule out some geographical differences which would affect representativeness of our study. However, patients were infected by all HCV genotypes with a distribution similar to that of the population in our environment [55], in the different stages of the disease, treated with all available combinations of DAA and without excluding patients with comorbidity. The indication and choice of treatment was based on the same protocol, developed according to clinical and efficiency criteria. HRQoL was analysed including subpopulations of patients usually underrepresented in clinical trials, such as those with psychiatric disorders or persons co-infected with HIV [23], which represent one third of the population in this study. All the interviews were carried out by the same investigator, in order to minimize the potential bias of the interviewer. The timing of the interview may affect the results. The post-12 interview was conducted in our study before knowing the SVR result, in order to avoid the possible overestimation on patient’s HRQoL self-assessment secondary to the euphoria experienced at that moment [43,56]. However, when patients know HCV become negative after treatment, the worry about the complications of the disease probably decrease and an improvement in the anxiety dimension could be greater than that observed in our study. A long-term evaluation of HRQoL after SVR would contribute to understand the effect of timing on HRQoL results.

Two possible shortcomings of our study need to be considered. First, the population size, although similar or greater than that of other observational studies [35,14,57] was not large enough to obtain conclusive results in some subanalyses. In any case, the patients included accounted for 78% of the patients who received treatment successfully during the study period. Second, the EQ-5D-5L is a generic questionnaire. Although it has been used in many scenarios, including chronic hepatitis C and its treatment, its combination with a disease-specific questionnaire would have been desirable. Time-related limitations in terms of patient care in the consulting room and of personnel related to completing the interviews discouraged the use of other questionnaires. However, studies that use both types of tools for measurement of patient reporting outcomes in patients treated with DAA therapy obtained similar results and good correlation between the generic and HCV-specific questionnaires [10,35]. EQ-5D-5L version was used in this study, as it has demonstrated to be a valid extension of EQ-5D-3L, providing more precise measurement at both individual and group level [58–61]. Since no 5L value sets were yet available in Spain, the crosswalk value set was used in this study, as the EuroQol recommended [31].

The new antivirals available recently seem to improve even more the treatment outcomes, being expected this same effect on quality of life.

## Conclusions

In summary, this study shows a short-term positive impact of SVR on the HRQoL of patients with chronic hepatitis C treated with interferon-free DAA. The greatest impact was on health dimensions of mobility, pain/discomfort, anxiety/depression, and in utility values and the VAS of the EQ-5D-5L questionnaire. The treatment did not produce disutility, nor did the use of RBV. Predictors of greater improvements in HRQoL with SVR may be baseline depression, anxiety, moderate-advanced fibrosis and cirrhosis; which may help to give priority to patients for treatment. The results obtained provide the patient's perspective in the assessment of DAA and information on patient reporting outcomes to be incorporated in cost-utility studies.

## Supporting information

S1 Table Study database. (XLS)
